
JOURNAL INFORMATION
==============================
NLM Title Abbreviation: ScientificWorldJournal
Journal ID: ScientificWorldJournal
NLM Journal ID: 101131163
Journal ID: TSWJ

The Scientific World Journal

ISSN: 2356-6140
EISSN: 1537-744X
Publisher: Wiley

ARTICLE INFORMATION
==============================
PMCID: PMC5917398
PMID: 16832570
DOI: 10.1100/tsw.2006.136
Article ID: 153097
Article version: 1
Subjects: Directions in Science

Endothelin-A Receptor Blockade Inhibits the Effects of Hypoxia on the Newborn Lung Vasculature

Ambalavanan Namasivayam *
Division of Neonatology, Department of Pediatrics, University of Alabama at Birmingham, USA
*Namasivayam Ambalavanan: ambal@uab.eduAcademic Editor: Martin Goette

Publication date: 2006
Electronic publication date: 2006 Jun 17
Volume: 6
First page: 669
Last page: 670
Received 2006 May 19; Accepted 2006 Jun 2
Copyright: Copyright © 2006 Namasivayam Ambalavanan.
Copyright year: 2006
License: This is an open access article distributed under the Creative Commons Attribution License, which permits unrestricted use, distribution, and reproduction in any medium, provided the original work is properly cited.
License URL: https://creativecommons.org/licenses/by/3.0/

Keywords: Persistent fetal circulation, endothelin-1, pulmonary hypertension, hypoxia, animal model, bronchopulmonary dysplasia

==============================
Directions in Science
*TheScientificWorldJOURNAL* (2006) 6, 669–670
ISSN 1537-744X; DOI 10.1100/tsw.2006.136

TheScientificWorldJOURNAL
www.thescientificworld.com

### Endothelin-A Receptor Blockade Inhibits the Effects of Hypoxia on the Newborn Lung Vasculature

Namasivayam Ambalavanan

*Division of Neonatology, Department of Pediatrics, University of Alabama at Birmingham*

**E-mail**: ambal@uab.edu

*Received May 19, 2006; Accepted June 2, 2006; Published June 17, 2006*

---

**KEYWORDS:** Persistent fetal circulation, endothelin-1, pulmonary hypertension, hypoxia, animal model, bronchopulmonary dysplasia

---

Disorders such as persistent pulmonary hypertension of the newborn (PPHN) and bronchopulmonary dysplasia (BPD) each affect more than 10,000 newborn infants every year in the U.S. alone, resulting in considerable mortality and morbidity. A common feature of these disorders is abnormal remodeling of the pulmonary blood vessels after birth and elevated pulmonary arterial pressures. Certain congenital heart diseases with markedly increased pulmonary blood flow also have abnormal pulmonary vasculature and high pulmonary blood pressures. Pulmonary arterial pressures are high in the fetus, and normally decrease at birth with lung inflation and oxygenation. Chronic hypoxemia just before birth or in the neonatal period may lead to abnormal thickening of the pulmonary arteries and pulmonary hypertension.

A recent study published in *Pediatric Research*[1] showed that blockade of the endothelin-A receptor was able to prevent and partially reverse hypoxia-induced pulmonary arterial remodeling in a newborn animal model. Endothelin-1 (ET-1) is a 21-amino-acid polypeptide that mediates vasoconstriction through the ET-A receptor located primarily on vascular smooth muscle cells and vasodilation through ET-B receptors located on endothelial cells. It has been previously shown that ET-1 levels are increased in newborn infants with PPHN, and are correlated with disease severity and decline with resolution of disease[2,3].

A previous study[4] showed that blockade of the ET-A receptor was able to reduce acute hypoxia-induced pulmonary hypertension in piglets, which generated the hypothesis that ET-A blockade may also be effective in preventing and reversing the effects of longer-term hypoxia on the pulmonary vasculature. To test this hypothesis, a newborn mouse model of hypoxia-induced pulmonary arterial remodeling was developed by exposure of C57BL/6 mice from birth to 2 weeks of age to hypoxia (12% oxygen) in a controlled environment chamber, along with their dams. The newborn mice were injected daily with either vehicle (cottonseed oil) or a selective ET-A antagonist (BQ-610) from either birth (the prevention study) or from 6 days of age (the reversal study). The effects of hypoxia on the pulmonary vasculature were evaluated by morphometry of the small pulmonary arteries, and by evaluation of right ventricular hypertrophy (an index of pulmonary hypertension, which causes hypertrophy of the right ventricle). It was observed that chronic hypoxia exposure led to thickening of small pulmonary arteries (<100 µm diameter) and right ventricular hypertrophy. ET-A blockade was able to prevent the thickening of small pulmonary arteries and right ventricular hypertrophy when given from birth, and significantly reduced the

©2006 with author.
Published by TheScientificWorld, Ltd.; www.thescientificworld.com arterial thickening when given from 6 days of age. These results demonstrate that ET-1 acting via ET-A receptors is a mediator of chronic hypoxia-mediated pulmonary arterial remodeling, and suggests that ET-A blockers may have therapeutic relevance in disorders such as PPHN or BPD.

Selective ET-A blockers are already known as promising therapeutic agents for adults with pulmonary hypertension[5,6]. The animal study described here indicates that such agents may also be suitable for the treatment of sick neonates with pulmonary hypertension and abnormal pulmonary vascular remodeling. However, several hurdles remain before ET-A antagonists enter the therapeutic armamentarium of neonatologists. ET-A antagonists are essentially untested in human neonates to date, and side effects such as hepatic toxicity are a concern in neonates who normally have impairment of some hepatic functions in the postnatal period. Initially, pilot trials of ET-A antagonists in neonates and older infants are required, evaluating safety and physiologic efficacy. Subsequently, larger randomized clinical trials are required to demonstrate efficacy of these agents on outcomes such as survival, need for extracorporeal membrane oxygenation (ECMO), and long-term morbidity in infants with established pulmonary hypertension. It is possible that effects of ET-A antagonists may be synergistic with the benefits of inhaled nitric oxide and other established therapies for neonatal pulmonary hypertension, and may improve clinical outcomes for critically ill neonates.

### REFERENCES

1. Ambalavanan, N., Bulger, A., Murphy-Ullrich, J., Oparil, S., and Chen, Y.F. (2005) Endothelin-A receptor blockade prevents and partially reverses neonatal hypoxic pulmonary vascular remodeling. *Pediatr. Res.* **57**, 631–636.
2. Kumar, P., Kazzi, N.J., and Shankaran, S. (1996) Plasma immunoreactive endothelin-1 concentrations in infants with persistent pulmonary hypertension of the newborn. *Am. J. Perinatol.* **13**, 335–341.
3. Rosenberg, A.A., Kennaugh, J., Koppenhafer, S.L., Loomis, M., Chatfield, B.A., and Abman, S.H. (1993) Elevated immunoreactive endothelin-1 levels in newborn infants with persistent pulmonary hypertension. *J. Pediatr.* **123**, 109–114.
4. Ambalavanan, N., Philips, J.B., 3rd, Bulger, A., Oparil, S., and Chen, Y.F. (2002) Endothelin-A receptor blockade in porcine pulmonary hypertension. *Pediatr. Res.* **52**, 913–921.
5. Langleben, D., Brock, T., Dixon, R., Barst, R.; STRIDE-1 study group (2004) STRIDE 1: Effects of the selective ETA receptor antagonist, sitaxsentan sodium, in a patient population with pulmonary arterial hypertension that meets traditional inclusion criteria of previous pulmonary arterial hypertension trials. *J. Cardiovasc Pharmacol.* **44**, S80–S84.
6. Widlitz, A.C., Barst, R.J., and Horn, E.M. (2005) Sitaxsentan: a novel endothelin-A receptor antagonist for pulmonary arterial hypertension. *Expert Rev. Cardiovasc. Ther.* **3**, 985–991.

**This article should be cited as follows:**

Ambalavanan, N. (2006) Endothelin-A receptor blockade inhibits the effects of hypoxia on the newborn lung vasculature**.** *TheScientificWorldJOURNAL* **6**, 669–670. DOI 10.1100/tsw.2006.136.

### BIOSKETCH

**Dr. Namasivayam Ambalavanan** is on the faculty of the Division of Neonatology, Department of Pediatrics, of the University of Alabama at Birmingham. He is board certified in pediatrics and in neonatal-perinatal medicine. In addition to clinical service at the Regional Neonatal Intensive Care Unit at the University Hospital and the Neonatal Intensive Care Unit at the Children's Hospital of Alabama, he is also the Director of the Newborn Unit at Cooper Green Hospital. Dr. Ambalavanan's research interests include the effects of hypoxia on the developing lung, as well as on reduction of lung injury in preterm infants using vitamin A and retinoic acid. His NIH-funded research is on the role of matrix metalloproteinase-2 (MMP-2) in neonatal hypoxia-induced pulmonary vascular remodeling. The University of Alabama at Birmingham is a participating center of the multicenter Neonatal Research Network of the NICHD, and Dr. Ambalavanan also participates in many clinical trials on critically ill preterm as well as term infants.
